# Assessment of the onset of lotilaner (Credelio™) speed of kill of fleas on dogs

**DOI:** 10.1186/s13071-017-2474-0

**Published:** 2017-11-01

**Authors:** Daniela Cavalleri, Martin Murphy, Wolfgang Seewald, Jason Drake, Steve Nanchen

**Affiliations:** 1 Elanco Animal Health, Mattenstrasse 24a, 4058 Basel, Switzerland; 2 Elanco Animal Health, 2500 Innovation Way, Greenfield, IN 46140 USA

**Keywords:** Fleas, *Ctenocephalides felis*, Lotilaner, Credelio™, Speed of kill, Dog, Oral

## Abstract

**Background:**

Lotilaner (Credelio™) is the newest member of the novel isoxazoline chemical class to be developed to treat canine ectoparasitism. Administered orally, lotilaner is rapidly absorbed with peak blood levels occurring within 2 h post-treatment. A study was undertaken to determine the earliest onset of lotilaner’s efficacy against existing flea infestations.

**Methods:**

From 72 Beagles, 64 qualifying dogs were ranked in descending order of flea counts from a Day -8 infestation and placed into eight blocks. Within blocks, eight dogs were randomly allocated among eight groups: Groups 1 to 4 were treated orally with lotilaner, at as close as possible to the minimum dose rate of 20 mg/kg within 30 (± 5) minutes after feeding; Groups 5 to 8 were untreated controls. All dogs were infested with 100 ± 5 fleas on Day -2, and whole-body flea counts were completed at 30 min and one, two and 8 h after treatment. Efficacy calculations were based on arithmetic and geometric means if an adequate infestation (at least six of eight untreated dogs with a flea retention of ≥ 50%) was demonstrated in the equivalent control group.

**Results:**

Adequate infestations were established in all control groups. At 30 min and 1 h post-treatment, relative to the matching untreated control group, there were no significant reductions in mean flea counts in lotilaner-treated dogs, although moribund fleas were evident at 1 h post-treatment. At 2 h after treatment, compared with the equivalent control group, the geometric mean flea count reduction in the lotilaner group was 64.0% (*t*_(7)_ = 2.86, *P* = 0.0242). At 8 h after treatment, lotilaner efficacy was 99.6%. There were no treatment-related adverse events.

**Conclusion:**

This study demonstrates that lotilaner flavored chewable tablets are well tolerated and begin to kill fleas within 2 h of treatment, achieving 99.6% efficacy within 8 h. Lotilaner can therefore be used to quickly alleviate the flea irritation that arises from existing infestations.

**Electronic supplementary material:**

The online version of this article (10.1186/s13071-017-2474-0) contains supplementary material, which is available to authorized users.

## Background

From the time of launch of the spot-on products fipronil and imidacloprid in the mid-1990s, speed of kill (SOK) of existing flea infestations has been a desirable feature of any new flea control product. The importance of this feature has been attributed to the need to provide a dog with relief from the irritation of flea bites, for affirmation of a pet owner’s perception of a product’s performance, and to quickly eliminate the source of egg production [1]. Subsequent to the appearance of the spot-ons, orally administered treatments were shown to demonstrate faster activity, and nitenpyram and spinosad were both shown to be 100% effective within 4 h after administration [1–3].

More recently, orally administered isoxazolines have emerged as rapid-onset products, although as yet none appeared to match the high-efficacy, rapid SOK that has been established for the earlier oral products against existing flea populations. A study comparing sarolaner and afoxolaner demonstrated that both products showed >99% efficacy against existing infestations when counts were completed at eight and 12 h post-treatment [4]. Two other studies demonstrated that sarolaner, fluralaner and spinosad/milbemycin oxime combination were 100% effective at 8 h post-treatment [5, 6]. In a report of two studies, at 3 h post-treatment spinosad was 86% and 93% effective, when in the same studies an isoxazoline, afoxolaner, was 3% to 26% effective at the same time point [7].

Lotilaner is a new isoxazoline that has a rapid onset of activity against fleas and ticks which is sustained through 35 days after treatment. In a series of studies, lotilaner efficacy against existing flea infestations fleas ranged from 89.9% at 4 h to 100% at eight and 12 h post-treatment. Against challenges throughout the month following treatment, efficacy at 4 h remained at > 97%, at six and 8 h remained at > 99%, and at 12 h remained at 100% [8]. As lotilaner is rapidly absorbed and achieves peak blood levels within 2 h of treatment, there was interest in determining just how quickly fleas would begin to succumb to treatment [9]. An additional study was therefore designed to provide further insight into the onset of lotilaner’s SOK at very early timepoints after administration against existing *Ctenocephalides felis* infestations on dogs.

## Methods

Allocation of dogs to groups, lotilaner administration and the independent witnessing of these tasks were the responsibility of non-blinded personnel. These and additional personnel required for dosing procedures and oversight (for example dose calculation verification), were not involved in any other study procedures. After allocation of the dogs to study groups, personnel involved in all experimental procedures other than treatment were blinded to the group allocation.

### Study dogs and housing

Inclusion criteria for study dogs were: to have an appropriate temperament that would allow flea infestations and counts to be completed uneventfully; to be clinically healthy and not pregnant; to be free of any sign of flea allergy dermatitis; and to be at least 7 months old and weigh between 8.9 and 19.6 kg at the beginning of the acclimation period. Dogs could not have been treated with a long acting topical or systemic product with activity against fleas during the 12 weeks preceding Day 0 (the day of treatment), nor treated within the past 6 months with any product containing an isoxazoline.

Beagle dogs aged between 9 months and 9 years, and weighing from 9.2 to 18.2 kg were acclimatized for 9 days before the day of lotilaner administration (Day 0). Dogs were housed individually in cages with no physical contact possible between dogs. Cages were inside indoor animal units that were environmentally controlled for temperature, which ranged over the study period between 16.1 °C and 22.9 °C. A photoperiod of 12 h light: 12 h darkness was maintained using overhead fluorescent lamps. Dogs were fed a commercially available, age-appropriate diet (VetsBrands Premium adult maintenance dog food and Eukanuba puppy, medium breed) once daily at the manufacturer recommended rates, and water was provided in stainless steel bowls that were replenished at least twice daily. On Day -8, 72 dogs were each infested with approximately 100 fleas. Dogs were combed 24 h later and flea counts were performed. The 64 qualifying dogs with the highest flea counts were selected for inclusion in the study.

### Treatment

The 64 dogs included in the study were ranked in descending order of individual flea counts from the Day -8 infestation and blocked into eight blocks, each of eight dogs, and within blocks randomly allocated among the eight groups. Groups 1 to 4 were treated orally with lotilaner as close as possible to the minimum recommended dose rate of 20 mg/kg within 30 (± 5) minutes after feeding, and Groups 5 to 8 were untreated controls. Each treated dog was observed immediately after administration and at 30 (± 5) minutes and 1 h (± 10 min) post-dosing by unblinded personnel. One regurgitated tablet was found in the passage leading to the cages from the examination rooms where lotilaner was administered. There was, however, no way to identify the dog from which this tablet came, and no dogs were re-dosed. No other incidents of tablets being spat out, regurgitated, or dogs vomiting were observed. Dogs in the untreated control group were removed from their cages and placed onto the dosing table as a sham treatment to maintain similar handling and to provide a reference time for post-treatment activities. Health observations were completed for all dogs at least once daily.

### Flea infestations and counts

Each dog was infested with approximately 100 adult unfed fleas of mixed sex on Days -8 (for selection and randomization) and -2. The fleas used for all infestations were from a laboratory bred colony (US strain) of *Ctenocephalides felis*. Flea counts and removals were completed as follows: Groups 1 and 5 at 30 min post-treatment; Groups 2 and 6 at 1 h post-treatment; Groups 3 and 7 at 2 h post-treatment; Groups 4 and 8 at 8 h post-treatment.

To recover fleas, each dog was combed for at least 5 min using strokes of the comb over each aspect of the dog’s body, each time moving in the same direction, following the pattern of the hair coat. Movement, from one part of the dog’s coat to the next, was via strokes overlapping each other, so that no area was missed. The procedure was then repeated until all dogs had been completely combed at least twice. If fleas were still present after the second full-body combing, the procedure was again repeated until no live or moribund fleas were found.

Recovered fleas were classified as live, moribund or dead. A flea was considered live if it could actively move through hair and if placed on a flat surface, rapidly righted itself and readily moved or jumped. A moribund flea was a flea that was laterally recumbent, could not normally move through hair or right itself when placed on a flat surface, but still had leg movement or twitching. A dead flea was a completely immobile flea.

### Efficacy assessment

Lotilaner efficacy against fleas at each time point was calculated according to the formula: Efficacy (%) = 100 × (Mc – Mt)/Mc, where Mc is the mean number of live fleas in the untreated control group (groups 5 to 8) on Day 0 and Mt is the mean number of live fleas in the corresponding lotilaner group (Groups 1 to 4) on Day 0. Efficacy calculations were based on arithmetic and geometric means. Geometric means were calculated using the flea (count +1) data and one (1) was subsequently subtracted from the result.

The time-point when lotilaner was considered to begin killing fleas was the first point at which there was a statistically significant decrease in geometric mean live flea counts, relative to the untreated control, as long as there was increasing efficacy, ultimately reaching at least 90%. Lotilaner was considered effective at a given time point if an adequate infestation was achieved in the control group (at least 50% retention of fleas in at least six dogs) at a given time-point and if there was a statistically significant difference (alpha = 0.05) in flea counts between the two groups, with a significant decrease in live fleas in the treated group compared to the control group. The statistical unit was the individual dog.

### Translations

Spanish translation of the article is available in Additional file 1. French translation of the Abstract is available in Additional file 2.

## Results

The administered lotilaner dose rates ranged from 20.5 to 29.1 mg/kg. However, the highest dose (in a Group 1 dog - 30 min post-treatment) was greater than targeted, and so this dog was excluded from calculations so as to not bias the results towards any increased speed of kill related to higher doses. Among the remaining dogs, the maximum dose rate was 24.7 mg/kg. Adequacy of infestation in the control groups (Groups 5 to 8) was achieved at all assessment time points, as more than six of the eight dogs in each had a flea retention of ≥ 50%, and mean live flea counts ranged from 76.9 to 88.8.

Although mean flea counts in the lotilaner group were numerically lower than in the control group at 30 min after treatment, there was no significant difference from the control group (*t*_(6)_ = 1.84, *P* = 0.1152), and only one moribund flea was found on one treated dog (Table 1). At 1 h post-treatment, there were no reductions in mean flea counts in lotilaner-treated dogs. At this assessment, 22 moribund fleas were collected from five of the lotilaner-treated dogs, and one moribund flea was found on a single dog in the control group (Table 1). At 2 h after treatment, compared with the matching control group, the geometric mean flea count reduction in the lotilaner group was 64.0% (*t*_(7)_ = 2.86, *P* = 0.0242) (arithmetic mean count reduction was 50.3%) (Table 2). This calculation includes the only treated dog after the two-hour assessment to have 100 fleas, none of which were moribund, while a total of 105 moribund fleas were removed from the other seven dogs in the group. At 8 h after treatment on Day 0, lotilaner efficacy based on geometric means was 99.6% (*P* < 0.0001), no fleas were classified as moribund and all recovered fleas were dead. At this point, the arithmetic mean flea count reduction was 98.8%.

**Table 1:** Day 0 flea counts of untreated control and lotilaner-treated dogs with moribund fleas counted as dead or included in live flea counts (moribund fleas counted as alive)

		Time post-treatment			
		30 min	1 h	2 h	8 h
Control Group					
Moribund fleas counted as dead	Range	78–100	45–100	34–100	57–100
	Arithmetic mean ± SD	88.8 ± 9.5	76.5 ± 17.9	79.0 ± 22.4	82.5 ± 14.1
	Geometric mean	88.3	74.5	75.2	81.4
Moribund fleas counted as alive	Range	78–100	46–100	34–100	57–100
	Arithmetic mean ± SD	88.8 ± 9.5	76.9 ± 17.7	79.1 ± 22.5	82.9 ± 14.3
	Geometric mean	88.3	74.9	75.4	81.7
Lotilaner Group					
Moribund fleas counted as dead	Range	41–81	72–97	9–100	0–8
	Arithmetic mean ± SD	77.3 ± 21.0	81.5 ± 8.3	39.3 ± 35.6	1.0 ± 2.8
	Geometric mean	74.5	81.2	27.1	0.3
Moribund fleas counted as alive	Range	42–100	75–100	31–100	0–8
	Arithmetic mean ± SD	77.4 ± 20.7	84.3 ± 8.2	52.4 ± 26.0	1.0 ± 2.8
	Geometric mean	74.7	83.9	48.0	0.3

*Abbreviation*: *SD* standard deviation

**Table 2:** Percent reduction in mean flea counts in lotilaner-treated dogs, with moribund fleas counted as dead or included in live flea counts (moribund fleas counted as alive)

	Time post-treatment			
	30 min	1 h	2 h	8 h
Moribund fleas counted as dead				
Arithmetic mean	12.9	0	50.3	98.8
Geometric mean	15.7	0	64.0	99.6
Comparison of groups	*t*_(6)_ = 1.84, *P* = 0.1152	*t*_(7)_ = -0.88, *P* = 0.4077	*t*_(7)_ = 2.86, *P* = 0.0242	*t*_(7)_ = 16.41, *P* < 0.0001
Moribund fleas counted as alive				
Arithmetic mean	12.8	0	33.8	98.8
Geometric mean	15.4	0	36.3	99.6
Comparison of groups	*t*_(6)_ = 1.86, *P* = 0.1123	*t*_(7)_ = -1.19, *P* = 0.2717	*t*_(7)_ = 2.26, *P* = 0.0583	*t*_(7)_ = 16.37, *P* < 0.0001

Other than for observations of dry skin and scales that developed in two treated dogs and two control dogs, there were no adverse events. These signs, which were also observed in four different study dogs prior to treatment, were attributed to the combination of flea infestations and flea combing.

## Discussion

**Fig. 1:**
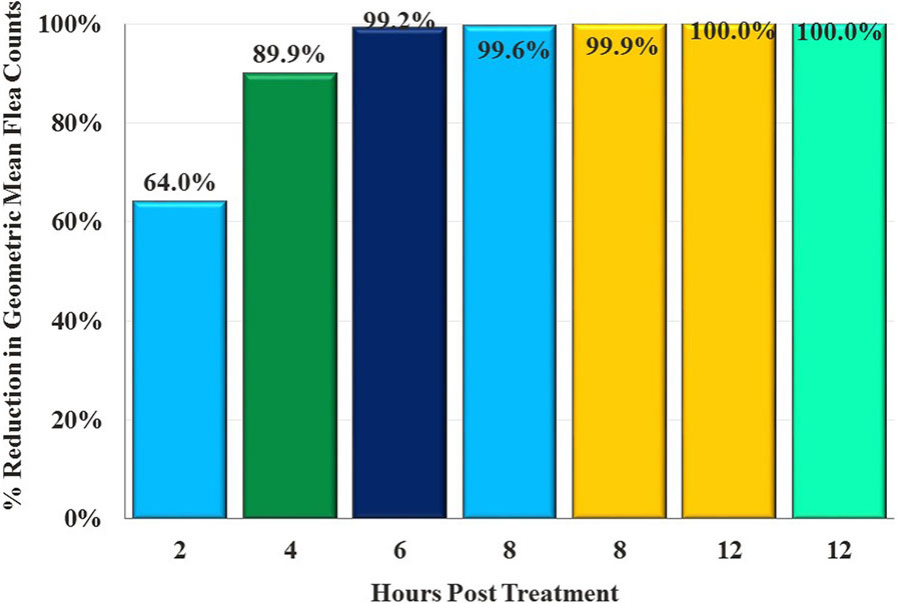
Composite of five different studies showing lotilaner efficacy against fleas, based on geometric means of treated dogs compared with untreated controls on the day of treatment. Columns in the same color represent a single study. The current study is in blue at 2 and 8 h; another study assessed the 6-h time-point, and one study assessed the 8-h time-point; two studies assessed the 12-h time-point [8]

A pattern of the rapid flea killing activity of lotilaner emerges when the results of this study are assessed within the context of other studies. In this study, at 1 h post-treatment the observation that 22 fleas in the lotilaner group were moribund is suggestive of an early onset of lotilaner efficacy. By 2 h post-treatment the numbers of moribund fleas had increased substantially, and there were significantly fewer live fleas in treated dogs (efficacy 64.0%; *t*_(7)_ = 2.86, *P* = 0.0242). In a separate study, by 4 h post-treatment overall reductions in live flea counts relative to untreated controls were 89.9%, and because of the high flea mortality at this point there were only low numbers of moribund fleas present [5]. In an additional study, by 6 h post-treatment mean reductions in live flea counts were 99.2%, respectively (Fig. 1) [8].

This activity aligns with the rapid absorption of lotilaner reported from a pharmacokinetic study in which detectable blood levels were identified in most treated dogs within 30 min after oral administration. Peak blood concentrations were then achieved within approximately 2 h after treatment [9]. Thus, almost immediately following dose administration any flea that feeds would be quickly exposed to lotilaner.

In the lotilaner two-hour group, it seems likely that the one dog with 100 live fleas, none of which were moribund, was responsible for the regurgitated tablet found immediately after treatment. However, because this could not be definitively shown, this dog’s flea count was included in the group analysis. Despite the inclusion of this dog’s data, the percent reduction in geometric mean flea counts compared to the control group (64.0%) was significant (*P* = 0.0242) (arithmetic mean reduction was 50.3%).

These results align with a report of four other studies in which efficacy against existing flea infestations was 89.9% at 4 h post-treatment, 99.2% at 6 h, 99.9% at 8 h post-treatment and 100% at 12 h (Fig. 1) [5]. In the study in which assessments were completed at 4 h post-treatment and post subsequent infestations, the SOK was sustained at greater than 99% through 1 month. In two studies efficacy was 100% at 12 h post-treatment and at every post-treatment challenge through Day 35. This sustained lotilaner speed of flea kill can reduce or eliminate irritation that might occur with subsequent reinfestations, and will result in flea death before egg-laying begins.

In the design of both the protocol and statistical analysis plan for this study, the authors were conscious of changing approaches on the part of regulatory authorities in Europe and the United States with regard to the issue of whether moribund fleas should be counted as live or dead [10]. We have presented both formats, but in efficacy assessments have counted moribund fleas as dead for two reasons. First, by definition moribund indicates the fleas were incapable of feeding and, regardless of whether or not they remained on the dog would have died. Second, if the moribund fleas had fallen off a treated dog they would not have been in a fit state to infest the same or a different host. Infestations of a host occur when immature stages in an environment emerge to find a new host. Only a very small proportion of fleas transfer between animals [11], and there is no record of fleas that have fallen from one host then being able to locate and infest a new host, particularly if those fleas had been in a moribund state at the time of displacement from the initial host.

## Conclusions

This study demonstrates that lotilaner was well tolerated and begins to kill fleas within 2 h of treatment, thereby being the first of the isoxazoline products to approximate the SOK of spinosad. This rapid onset of flea-killing should quickly lead to alleviation of the flea irritation that arises from existing infestations.

## Additional files

Additional file 1: Spanish translation of the article. (PDF 77 kb)

Additional file 2: French translation of the Abstract. (PDF 53 kb)

## Abbreviations

SOK: speed of kill
