# Supplementary material for: Assessment of the onset of lotilaner (Credelio™) speed of kill of fleas on dogs
Source: Parasit Vectors. 2017 Nov 1;10:521. doi: 10.1186/s13071-017-2474-0 (PMC5664436; doi:10.1186/s13071-017-2474-0)
Supplement: Supplementary file 1 — Spanish translation of the article. (PDF 77 kb) [file 13071_2017_2474_MOESM1_ESM.pdf]

Evaluación de la velocidad del inicio para matar pulgas en perros de lotilaner (Credelio™)

Daniela Cavalleri[1], Martin Murphy[1], Wolfgang Seewald[1], Jason Drake[2]*, Steve Nanchen[1]

[1]Elanco Animal Health, Schwarzwaldallee 215, CH-4058 Basel, WRO-1032.2.58, Suiza
[2]Elanco Animal Health, 2500 Innovation Way, Greenfield, IN 46140, USA

*Correspondencia: drake_jon_j@elanco.com

Emails:
Daniela Cavalleri: cavalleri_daniela_a@elanco.com
Martin Murphy: murphy_martin_gerard@elanco.com
Wolfgang Seewald: seewald_wolfgang@elanco.com
Jason Drake: drake_jon_j@elanco.com
Steve Nanchen: nanchen_steve@elanco.com

Resumen

Antecedentes: Lotilaner (Credelio™) es el miembro más nuevo de la clase química de las isoxazolinas desarrollado para tratar el ectoparasitismo canino. Lotilaner se administra oralmente y se absorbe rápidamente, alcanzando niveles pico en sangre que ocurren al cabo de dos horas post-tratamiento. Se llevó a cabo un estudio para determinar el inicio más temprano de la eficacia de lotilaner contra las infestaciones existentes de pulgas.

Métodos: De 72 Beagles, 64 perros que calificaron se clasificaron en orden descendiente de acuerdo al conteo de pulgas del Día-8 de infestación y se colocaron en ocho bloques. Dentro de los bloques, ocho perros se ubicaron aleatoriamente entre los ocho grupos: Los grupos 1 a 4 se trataron oralmente con lotilaner, tan cercanamente posible al nivel de dosis mínima de 20 mg/kg al cabo de 30 (± 5) minutos después de su alimentación; los grupos 5 a 8 fueron controles no tratados. Todos los perros se infestaron con 100 ± 5 pulgas en el Día -2, los conteos de pulgas del cuerpo completo se terminaron en 30 minutos y una, dos y ocho horas después del tratamiento. Los cálculos de

eficacia se basaron en medias aritméticas y geométricas si una infestación adecuada (al menos seis de ocho perros no tratados con una retención de pulgas de ≥ 50%) era demostrado en el grupo control equivalente.

Resultados: Se establecieron las infestaciones adecuadas en todos los grupos control. A 30 minutos y una hora post-tratamiento, en relación con la coincidencia del grupo control no tratado, no hubo reducciones significativas en las medias del conteo de pulgas en los perros tratados con lotilaner, aunque las pulgas moribundas fueron evidentes a una hora post-tratamiento. A dos horas después del tratamiento, comparado con el grupo control equivalente, la media geométrica de la reducción en el conteo de pulgas del grupo de lotilaner fue del 64.0% ($t_{(7)} = 2.86$, $P = 0.0242$). A las ocho horas después del tratamiento, la eficacia de lotilaner fue del 99.6%. No hubo eventos adversos relacionados con el tratamiento.

Conclusión: Este estudio demostró que las tabletas masticables saborizadas de lotilaner son bien toleradas y comienzan a matar pulgas al cabo de dos horas del tratamiento, logrando una eficacia del 99.6% al cabo de ocho horas. Por lo tanto, lotilaner puede ser utilizado para aliviar rápidamente la irritación por pulgas que surge de las infestaciones existentes.

Palabras clave: Pulgas, *Ctenocephalides felis*, Lotilaner, Credelio™, Velocidad para matar, Perro, Oral

Antecedentes

Desde el lanzamiento de los productos spot-on de fipronil y de imidacloprid a mediados de los 90’s, la velocidad para matar (SOK, por sus siglas en inglés) de las infestaciones por pulgas ha sido una característica deseable de cualquier nuevo producto de control de pulgas. La importancia de esta característica se ha atribuido a la necesidad de proveer a un perro con el alivio de la irritación causada por las mordeduras de pulgas, por la afirmación de la percepción del dueño de la mascota del desempeño del producto, y eliminar rápidamente la fuente de la producción de huevos [1]. Subsecuente a la aparición de los spot-ons, los tratamientos administrados oralmente se probaron para demostrar una actividad más rápida, y tanto nitenpyram y el spinosad demostraron ser 100% efectivos al cabo de cuatro horas después de su administración [1-3].

Más recientemente, las isoxazolinas administradas oralmente surgieron como los productos con el arranque o inicio más rápido, aunque hasta ahora ninguno aparece coincidir con alta-eficacia, y rápida SOK que haya sido establecida par los productos orales anteriores contra las poblaciones

existentes de pulgas. Un estudio comparando sarolaner y afoxolaner demostraron que ambos productos muestran un > 99% de eficacia contra las infestaciones existentes cuando los conteos se completaron a las ocho y a las 12 horas post-tratamiento [4]. Otros dos estudios demostraron que sarolaner, fluralaner y la combinación de spinosad/milbemicina oxima fueron 100% efectivas a las 8 horas post-tratamiento [5,6]. En un reporte de dos estudios, a las tres horas post-tratamiento spinosad era 86% y 93% efectivo, cuando en los mismos estudios una isoxazolina, afoxolaner, fue 3% a 26% efectivo en el mismo punto en el tiempo [7].

Lotilaner es una nueva isoxazolina que tiene un rápido inicio de su actividad contra pulgas y garrapatas que es sostenida a lo largo de 35 días después del tratamiento. En una serie de estudios, la eficacia de lotilaner contra las infestaciones existentes de pulgas tenía un rango de 89.9% a las cuatro horas a 100% a las ocho y 12 horas post-tratamiento. Contra los desafíos a lo largo del mes después del tratamiento, la eficacia a las cuatro horas permaneció a > 97%, a las seis y ocho horas y se mantuvo a > 99%, y a las 12 horas permaneció a 100% [8]. Como lotilaner se absorbe rápidamente y logra los niveles pico en sangre al cabo de dos horas de tratamiento, hubo interés en determinar que tan rápido comenzarían las pulgas a sucumbir ante el tratamiento [9]. Por lo tanto, se diseñó un estudio adicional para proveer una mayor percepción en el en el inicio de la SOK de lotilaner en puntos en el tiempo muy tempranos después de la administración contra las infestaciones existentes por *Ctenocephalides felis* en perros.

Métodos

La ubicación de los perros a los grupos, la administración de lotilaner y el testimonio independiente de estas tareas fue la responsabilidad de personal no-ciego. Este personal y personal adicional requirieron de procedimientos de dosificación y vigilancia (por ejemplo, la verificación del cálculo de la dosis), no estuvieron involucrados en ningún otro procedimiento del estudio. Después de la ubicación de los perros a los grupos de estudio, el personal involucrado en todos los procedimientos experimentales más que de tratamiento fueron ciegos a la ubicación del grupo.

Perros de estudio y alojamiento

El criterio de inclusión para los perros de estudio fueron: tener un temperamento apropiado que le permitiera las infestaciones de pulgas y que los conteos se completaran sin problemas; estar clínicamente sanos y no gestantes; estar libres de cualquier signo de dermatitis alérgica al piquete de

pulga; y tener al menos siete meses de edad y pesar entre 8.9 y 19.6 kg al inicio del período de aclimatización. Los perros no podían haber sido tratados con un producto tópico o sistémico de larga duración con actividad contra pulgas durante las 12 semanas anteriores al Día 0 (el día de tratamiento), ni ser tratados al cabo de los últimos seis meses con ningún producto que contuviera una isoxazolina.

Los perros Beagle tenían entre nueve meses y nueve años de edad, pesando de 9.2 a 18.2 kg y se aclimatizaron por nueve días antes del día de la administración de lotilaner (Día 0). Los perros fueron alojados individualmente en jaulas sin contacto físico posible entre ellos. Las jaulas estaban dentro de las unidades interiores para animales con ambiente controlado para la temperatura, la cuál tenía un rango en el período de estudio entre 16.1 °C y 22.9 °C. Un fotoperiodo de 12 horas luz: 12 horas de oscuridad se mantuvieron usando lámparas fluorescentes suspendidas. Los perros fueron alimentados con una dieta comercialmente disponible apropiada para la edad (VetsBrands Premium alimento de mantenimiento para adultos y Eukanuba puppy, raza intermedia) una vez al día al nivel recomendado por el fabricante, y agua administrada en tazones de acero inoxidable que se rellenaban al menos dos veces al día. En el Día -8, 72 perros fueron infestados aproximadamente con 100 pulgas. Los perros se peinaron 24 horas después y se llevaron a cabo los conteos de las pulgas. Los 64 perros que calificaron con los conteos más altos de pulgas se seleccionaron para su inclusión en el estudio.

Tratamiento

Los perros incluidos en el estudio se clasificaron en orden descendiente de acuerdo al conteo individual de pulgas en el Día -8 de la infestación y fueron colocados en 8 bloques, cada uno de ocho perros, y dentro de cada bloque se ubicaron aleatoriamente entre los ocho grupos. Los grupos 1 a 4 fueron tratados oralmente con lotilaner lo más cercano posible al nivel de la dosis mínima de 20 mg/kg al cabo de 30 (± 5) minutos después de la alimentación, y los Grupos 5 y 8 fueron los controles no tratados. Cada perro tratado se observó inmediatamente después de la administración y a los 30 (± 5) minutos y 1 hora (± 10 minutos) post-dosificación por personal no ciego. Se encontró una tableta regurgitada en el pasillo que lleva a las jaulas de las salas de examinación en donde se administró lotilaner. Sin embargo, no hubo manera de identificar al perro del cuál provino esta tableta y ningún perro se re-dosificó. No hubo otros incidentes observados de tabletas escupidas o regurgitadas o perros vomitando. Los perros en el grupo control no tratado fueron sacados de sus jaulas y colocados en la mesa de dosificación como un tratamiento simulado para mantener un

manejo similar y proveer un tiempo de referencia para las actividades post-tratamiento. Las observaciones de salud se completaron para todos los perros al menos una vez al día.

Infestaciones de pulgas y conteos

Cada perro se infestó con aproximadamente 100 pulgas adultas no alimentadas de ambos sexos en el Días -8 (para la selección y aleatorización) y en el -2. Las pulgas usadas para todas las infestaciones fueron de una colonia criada en laboratorio (cepa de E.U.A.) de *Ctenocephalides felis*. Los conteos de pulgas y las remociones se completaron como sigue:: Grupos 1 y 5 a 30 minutos post-tratamiento; Grupos 2 y 6 a 1 hora post-tratamiento; Grupos 3 y 7 a las 2 horas post-tratamiento; Grupos 4 y 8 a las 8 horas post-tratamiento.

Para recuperar las pulgas, cada perro se peinó por al menos cinco minutos usando golpes de peine sobre cada aspecto del cuerpo del perro, cada vez moviéndolo en la misma dirección, siguiendo el patrón del pelaje. El movimiento, de una parte del pelaje del perro al siguiente fue por la vía de traslapar los golpes del peine entre cada uno, para que no se perdiera ninguna área. Luego se repitió el procedimiento hasta que todos los perros se peinaron completamente al menos dos veces. Sí las pulgas todavía estaban presentes después de la segunda peinada de todo el cuerpo, el procedimiento se volvía a repetir hasta que no se encontraran pulgas vivas o moribundas.

Las pulgas se clasificaron como vivas, moribundas o muertas. Una pulga se consideraba viva si podía moverse activamente a través del pelo y sí se colocaba en una superficie plana, rápidamente se enderezaba y rápidamente se movía o brincaba. Una pulga moribunda era una pulga en decúbito lateral, que no se podía mover normalmente a través del pelo o enderezarse sola cuando se colocaba en una superficie plana, pero todavía seguía teniendo movimientos de las patas o espasmos. Una pulga muerta era una pulga completamente inmóvil.

Evaluación de eficacia

La eficacia de lotilaner contra las pulgas en cada punto en el tiempo se calculó de acuerdo a la fórmula: Eficacia (%) = 100 × (Mc – Mt)/Mc, en dónde Mc es la media del número de pulgas vivas en el grupo control no tratado (grupos 5 a 8) en el Día 0 y Mt es la media del número de pulgas vivas en el grupo correspondiente a lotilaner (Grupos 1 a 4) en el Día 0. Los cálculos de eficacia se basaron en medias aritméticas y geométricas. Las medias geométricas se calcularon usando los datos de pulgas (conteo + 1) y uno (1) se restaba del resultado subsecuentemente.

El punto en el tiempo cuando lotilaner se consideró que iba a matar a las pulgas fue el primer punto en el que hubo un decremento estadísticamente significativo en la media geométrica de los

conteos de las pulgas vivas, en relación con el control no tratado, siempre que hubiera un aumento en la eficacia, finalmente alcanzando al menos un 90%. Lotilaner se consideró efectivo en un punto dado en el tiempo si una infestación adecuada se lograba en el grupo control (al menos un 50% de la retención de las pulgas en al menos seis perros) en un punto dado en el tiempo y sí hubo una diferencia estadísticamente significativa (alfa = 0.05) en el conteo de pulgas entre dos grupos, con un decremento significativo en las pulgas vivas en el grupo tratado en comparación con el grupo control. La unidad estadística fue el perro individual.

Resultados

Los niveles de dosis administrados de lotilaner estuvieron en un rango de 20.5 a 29.1 mg/kg. Sin embargo, la dosis más elevada (en el Grupo 1 perro – 30 minutos post-tratamiento) fue mayor que el objetivo, y entonces este perro fue excluido de los cálculos para no tener sesgos en los resultados hacia algún incremento en la velocidad de matar en relación con las dosis más elevadas. Entre los perros restantes, el nivel de dosis máxima fue de 24.7 mg/kg. La adecuación de la infestación en los grupos control (Grupos 5 a 8) se logró en todos los puntos de evaluación, como más de seis de ocho perros en cada retención de pulgas de ≥ 50%, y los conteos de pulgas vivas tenían un rango de 76.9 a 88.8.

Aunque las medias de los conteos de pulgas en el grupo de lotilaner fueron numéricamente más bajos que en el grupo control a los 30 minutos después del tratamiento, no hubo una diferencia significativa con el grupo control ($t_{(6)} = 1.84$, $P = 0.1152$), y solamente se encontró una pulga moribunda en uno de los perros tratados (Tabla 1). A la hora post-tratamiento, no hubo reducciones en las medias de los conteos de pulgas en los perros tratados con lotilaner. En esta evaluación, 22 pulgas moribundas se colectaron de cinco de los perros tratados con lotilaner, y una pulga moribunda se encontró en un solo perro en el grupo control (Tabla 1). A las dos horas después del tratamiento, en comparación con el grupo control correspondiente, la reducción en la media geométrica del conteo de pulgas en el grupo de lotilaner fue de 64.0% ($t_{(7)} = 2.86$, $P = 0.0242$) (reducción de la media aritmética de 50.3%) (Tabla 2). Este cálculo incluye solo al perro tratado después de dos horas de evaluación para tener 100 pulgas, ninguna de las cuales estaba moribunda, mientras que un total de 105 pulgas moribundas se removieron de otros siete perros en el grupo. A las ocho horas después del tratamiento en el Día 0, la eficacia de lotilaner basada en las medias geométricas fue de 99.6% ($P < 0.0001$), ninguna pulga se clasificó como moribunda y todas las

pulgas recuperadas estaban muertas. En este punto, la reducción en la media aritmética del conteo de pulgas fue del 98.8%.

No hubo eventos adversos, solamente observaciones de piel seca y escamas que se desarrollaron en dos perros tratados y en dos perros control. Estos signos, que también se observaron en cuatro perros en diferente estudio previo al tratamiento, se atribuyen a la combinación de las infestaciones por pulgas y al peinado.

Discusión

Un patrón de la actividad rápida para matar pulgas de lotilaner surge cuando los resultados de este estudio se evalúan dentro del contexto de otros estudios. En este estudio, a una hora post-tratamiento la observación de que 22 pulgas en el grupo de lotilaner estaban moribundas sugiere un inicio temprano de la eficacia de lotilaner. Para las dos horas post-tratamiento los números de las pulgas moribundas se había incrementado substancialmente, y hubieron, significativamente, menos pulgas vivas en los perros tratados (eficacia 64.0%; $t_{(7)} = 2.86$, $P = 0.0242$). En un estudio separado, para las cuatro horas post-tratamiento las reducciones generales en el conteo de pulgas vivas en relación con los controles no tratados fueron del 89.9%, y debido a la alta mortalidad en este punto solo hubo números bajos de pulgas moribundas presentes [5]. En un estudio adicional, para las seis horas post-tratamientos las reducciones en las medias de los conteos de las pulgas vivas fueron del 99.2%, respectivamente (Fig. 1) [8].

Esta actividad se alinea con la rápida absorción de lotilaner reportada en un estudio de farmacocinética en el que se identificaron niveles detectables en sangre en la mayoría de los perros tratados al cabo de 30 minutos después de la administración oral. Las concentraciones pico en sangre se lograron al cabo de dos horas después del tratamiento aproximadamente [9]. Por ende, casi inmediatamente después de la administración de la dosis cualquier pulga que se alimentara se expondría rápidamente a lotilaner.

En el grupo de lotilaner de dos-horas, parece que el perro con 100 pulgas vivas, ninguna de ellas moribundas, fue el responsable de haber regurgitado la tableta que se encontró inmediatamente después del tratamiento. Sin embargo, debido a que esto no podía ser definitivamente demostrado, el conteo de pulgas de este perro se incluyó en el grupo de análisis. A pesar de la inclusión de los datos de este perro, la reducción porcentual de la media geométrica del conteo de pulgas comparado con el grupo control (64.0%) fue significativa ($P = 0.0242$) (reducción de la media aritmética 50.3%).

Estos resultados se alinean con un reporte de otros cuatro estudios en los que la eficacia contra las infestaciones existentes por pulgas era del 89.9% a las cuatro horas post-tratamiento, 99.2% a las seis horas, 99.9% a las ocho horas post-tratamiento y 100% a las 12 horas (Fig. 1) [5]. En el estudio en el cual las evaluaciones se completaron a las cuatro horas post-tratamiento y las infestaciones post-subsecuentes, la SOK se sostuvo a más de un 99% a lo largo de un mes. En dos estudios la eficacia fue del 100% a las 12 horas post-tratamiento y en cada desafío post-tratamiento hasta el Día 35. La velocidad sostenida para matar las pulgas de lotilaner puede reducir o eliminar la irritación que pueda ocurrir con las reinfestaciones subsecuentes, y resultará en la muerte de la pulga antes de que se inicie la ovoposición.

En el diseño de tanto el protocolo y el plan del análisis estadístico para este estudio, los autores estaban conscientes de cambiar enfoques en la parte de las autoridades regulatorias en Europa y Estados Unidos con respecto al asunto de si las pulgas moribundas se deberían de contar como vivas o muertas [10]. Hemos presentado ambos formatos, pero en las evaluaciones de eficacia se han contado las pulgas moribundas como muertas por dos razones. Primero, por definición moribunda indica que las pulgas fueron incapaces de alimentarse e, independientemente de si se quedaron en el perro o no hubieran muerto. Segundo, si las pulgas moribundas se caen del perro tratado no estarían en un estado adecuado para infestar al mismo hospedador o a uno diferente. Las infestaciones de un hospedador ocurren cuando las etapas inmaduras en un ambiente surgen para encontrar a un nuevo hospedador. Solamente una pequeña proporción de pulgas se transfiere entre animales [11], y no hay registro de pulgas que se hayan caído de un hospedador luego de haber sido localizadas e infestar a un nuevo hospedador, particularmente, si esas pulgas han estado en un estado moribundo al momento del desplazamiento del hospedador inicial.

Conclusiones

Este estudio demuestra que lotilaner fue bien tolerado y comienza a matar pulgas al cabo de dos horas del tratamiento, de ahí que siendo el primero de los productos de isoxazolina que se aproxima a la SOK del spinosad. Este rápido inicio para matar pulgas deberá de llevar rápidamente al alivio de la irritación causada por las pulgas que surge por las infestaciones existentes.

Abreviaturas

SOK: velocidad para matar

Reconocimientos

Los autores quieren agradecer al Dr. Bill Ryan de Ryan Mitchell Associates LLC por su apoyo con el manuscrito.

Declaraciones

Declaración de ética

Este estudio de laboratorio ciego, aleatorizado, con control negativo fue llevado a cabo de acuerdo con el protocolo, en cumplimiento con los principios de Buenas Prácticas Clínicas, y con la Asociación Mundial para el Avance de las Guías de Parasitología Veterinaria para evaluar la eficacia de los parasiticidas para el tratamiento, prevención y control de la infestación de pulgas y garrapatas en perros y gatos. El protocolo fue aprobado por el Comité de ClinVet para la Ética y Bienestar Animal. Las instalaciones fueron asignadas con la consideración del Estándar Nacional Sudafricano SANS 10386:2008 “cuidado y uso de los animales para propósitos científicos”.

Consentimiento para publicación

No aplicable.

Disponibilidad de datos y material

Todos los datos generados o analizados durante este estudio se incluyeron en este artículo publicado.

Fondos

El estudio fue patrocinado por Elanco.

Intereses competentes

DC, WS, MM, JD y SN son empleados de Elanco Animal Health.

Contribuciones de los autores

Todos los autores participaron en el diseño y finalización de los estudios y se involucraron en el manuscrito preliminar. Todos los autores leyeron y aprobaron el manuscrito final.

Detalles del autor

[1]Elanco Animal Health, Schwarzwaldallee 215, CH-4058 Basel, WRO-1032.2.58, Suiza.
[2]Elanco Animal Health, 2500 Innovation Way, Greenfield, IN 46140, EE.UU.

Referencias

1. Dryden MW, McCoy CM, Payne PA. Speed of flea kill with nitenpyram tablets compared to imidacloprid spot on and fipronil spot on in dogs. Compend Contin Educ Pract Vet. 2001;23(Suppl 3A):24–7.
2. McCoy C, Broce AB, Dryden MW. Flea blood feeding patterns in cats treated with oral nitenpyram and the topical insecticides imidacloprid, fipronil and selamectin. Vet Parasitol. 2008;156:293-301.
3. Blagburn BL, Young DR, Moran C, Meyer JA, Leigh-Heffron A, Paarlberg T, et al. Effects of orally administered spinosad (Comfortis) in dogs on adult and immature stages of the cat flea (*Ctenocephalides felis*). Vet Parasitol. 2010;168:312–7.
4. Six RH, Liebenberg J, Honsberger NA, Mahabir SP. Comparative speed of kill of sarolaner (Simparica) and afoxolaner (NexGard) against induced infestations of *Ctenocephalides felis* on dogs. Parasit Vectors. 2016;9:90.
5. Six RH, Liebenberg J, Honsberger NA, Mahabir SP. Comparative speed of kill of sarolaner (Simparica™) and fluralaner (Bravecto®) against induced infestations of *Ctenocephalides felis* on dogs. Parasit Vectors. 2016;9:92.
6. Six RH, Everett WR, Myers MR, Mahabir SP. Comparative speed of kill of sarolaner (Simparica™) and spinosad plus milbemycin oxime (Trifexis) against induced infestations of *Ctenocephalides felis* on dogs. Parasit Vectors. 2016;9:93.
7. Snyder DE, Rumschlag AJ, Young LM, Ryan WG. Speed of flea knockdown of spinosad compared to afoxolaner, and of spinosad through 28 days post-treatment in controlled laboratory studies. Parasit Vectors. 2015;8:578.
8. Cavalleri D, Murphy M, Seewald W, Drake J, Nanchen S. Assessment of the speed of flea kill of lotilaner (Credelio™) throughout the month following oral administration to dogs. Parasit Vectors. 2017 (In press).
9. Toutain CE, Seewald W, Jung M. The intravenous and oral pharmacokinetics of lotilaner and the effect of food in dogs. Parasit Vectors. (In press).
10. European Medicines Agency, Committee for Medicinal Products for Veterinary Use. Guideline for the testing and evaluation of the efficacy of antiparasitic substances for the treatment and prevention of tick and flea infestation in dogs and cats. 2016.

http://www.ema.europa.eu/docs/en_GB/document_library/Scientific_guideline/2016/07/WC500210927.pdf. Accessed January 31, 2017.

11. Rust MK, Dryden MW. The biology, ecology and management of the cat flea. Annu Rev Entomol. 1997:42:451-73.

Fig. 1 Gráfica integrada con cinco estudios diferentes que mostraban la eficacia de lotilaner contra las pulgas, basadas en medias geométricas de los perros tratados comparados con los controles no tratados en el día de tratamiento. Las columnas con el mismo color representan un solo estudio. El estudio actual está en azul a las 2 y 8 horas; otro estudio evaluado a las 6-horas como punto de tiempo, y un estudio evaluado a las 8 horas como punto de tiempo; dos estudios evaluados a las 12 horas como punto de tiempo [8]

Tabla 1 Conteos de pulgas en el Día 0 del control no tratado y de los perros tratados con lotilaner con pulgas moribundas contadas como muertas o incluidas en el conteo de pulgas vivas (pulgas moribundas contaban como vivas)

		Tiempo post-tratamiento			
		**30 minutos**	**1 hora**	**2 horas**	**8 horas**
Grupo Control					
Pulgas moribundas contadas como muertas	Rango	78–100	45–100	34–100	57–100
	Media aritmética ± DE	88.8 ± 9.5	76.5 ± 17.9	79.0 ± 22.4	82.5 ± 14.1
	Media geométrica	88.3	74.5	75.2	81.4
Pulgas moribundas contadas como vivas	Rango	78–100	46–100	34–100	57–100
	Media aritmética ± DE	88.8 ± 9.5	76.9 ± 17.7	79.1 ± 22.5	82.9 ± 14.3
	Media geométrica	88.3	74.9	75.4	81.7
Grupo de Lotilaner					
Pulgas moribundas contadas como muertas	Rango	41–81	72–97	9 - 100	0–8
	Media aritmética ± DE	77.3 ± 21.0	81.5 ± 8.3	39.3 ± 35.6	1.0 ± 2.8
	Media geométrica	74.5	81.2	27.1	0.3
Pulgas moribundas contadas como vivas	Rango	42–100	75–100	31–100	0–8
	Media aritmética ± DE	77.4 ± 20.7	84.3 ± 8.2	52.4 ± 26.0	1.0 ± 2.8
	Media geométrica	74.7	83.9	48.0	0.3

Abreviatura: DE, desviación estándar

Tabla 2 Reducción porcentual en la media de conteos de pulgas en los perros tratados con lotilaner, con pulgas moribundas contadas como muertas o incluidas en el conteo de pulgas vivas (pulgas moribundas contadas como vivas)

	**Tiempo post-tratamiento**			
	**30 minutos**	**1 hora**	**2 horas**	**8 horas**
Pulgas moribundas contadas como muertas				
Media aritmética	12.9	0	50.3	98.8
Media geométrica	15.7	0	64.0	99.6
Comparación de grupos	$t_{(6)} = 1.84$, $P = 0.1152$	$t_{(7)} = -0.88$, $P = 0.4077$	$t_{(7)} = 2.86$, $P = 0.0242$	$t_{(7)} = 16.41$, $P < 0.0001$
Pulgas moribundas contadas como vivas				
Media aritmética	12.8	0	33.8	98.8
Media geométrica	15.4	0	36.3	99.6
Comparación de grupos	$t_{(6)} = 1.86$, $P = 0.1123$	$t_{(7)} = -1.19$, $P = 0.2717$	$t_{(7)} = 2.26$, $P=0.0583$	$t_{(7)} = 16.37$, $P < 0.0001$
