# Supplementary material for: Assessment of the onset of lotilaner (Credelio™) speed of kill of fleas on dogs
Source: Parasit Vectors. 2017 Nov 1;10:521. doi: 10.1186/s13071-017-2474-0 (PMC5664436; doi:10.1186/s13071-017-2474-0)
Supplement: Supplementary file 2 — French translation of the Abstract. (PDF 53 kb) [file 13071_2017_2474_MOESM2_ESM.pdf]

Évaluation de la rapidité d'action du lotilaner (Credelio™) contre les puces chez le chien

Daniela Cavalleri[1], Martin Murphy[1], Wolfgang Seewald[1], Jason Drake[2]* et Steve Nanchen[1]

[1]Elanco Santé animale, Schwarzwaldallee 215, CH-4058 Bâle, WRO-1032.2.58, Suisse
[2]Elanco Santé animale, 2500 Innovation Way, Greenfield, IN 46140, États-Unis

*Correspondance : drake_jon_j@elanco.com

Adresses électroniques :
Daniela Cavalleri : cavalleri_daniela_a@elanco.com
Martin Murphy : murphy_martin_gerard@elanco.com
Wolfgang Seewald : seewald_wolfgang@elanco.com
Jason Drake : drake_jon_j@elanco.com
Steve Nanchen : nanchen_steve@elanco.com

Résumé

Contexte : le lotilaner (Credelio™) est le tout dernier agent de la nouvelle classe des isoxazolines à être développé dans la lutte contre les ectoparasites chez le chien. Après administration par voie orale, le lotilaner est rapidement absorbé, le pic de concentration plasmatique étant atteint dans les deux heures. Une étude a été menée afin de déterminer le délai minimal à l'issue duquel le lotilaner commence à être efficace contre les infestations existantes par les puces.

Méthodes : 64 chiens ont été sélectionnés parmi 72 beagles et classés par nombre décroissant de puces récoltées dans leur pelage suite à une infestation à J8 puis répartis dans huit blocs. Les huit chiens de chaque bloc ont été randomisés dans huit groupes : les groupes 1 à 4 ont reçu par voie orale une dose de lotilaner aussi proche que possible de la dose minimale de 20 mg/kg dans les 30 (± 5) minutes suivant un repas ; les groupes 5 à 8 étaient ceux des témoins non traités. Tous les chiens ont été infestés par 100 ± 5 puces le jour -2. Les puces ont ensuite été dénombrées sur l'ensemble du corps 30 minutes, une, deux et huit heures après l'administration du traitement.

L'efficacité était mesurée à partir des moyennes arithmétiques et géométriques si une infestation suffisante (persistance de ≥ 50 % des puces chez six des huit chiens non traités) était démontrée dans le groupe témoin correspondant.

Résultats : la présence d'infestations suffisantes a été établie pour tous les groupes témoins. A 30 minutes et 1 heure du traitement, aucune réduction significative du nombre de puces n'a été observée chez les chiens ayant reçu le lotilaner par rapport au groupe témoin correspondant. La présence de puces moribondes était néanmoins évidente une heure après le traitement. Deux heures après le traitement, la diminution (moyenne géométrique) du nombre de puces dans le groupe lotilaner était de 64,0 % ($t_{(7)} = 2,86$, $p = 0,0242$) par rapport au groupe témoin correspondant. Huit heures après le traitement, l'efficacité du lotilaner était de 99,6 %. Aucun événement indésirable lié au traitement n'a été observé.

Conclusion : cette étude démontre que les comprimés à croquer aromatisés à base de lotilaner sont bien tolérés et qu'ils commencent à tuer les puces dans les deux heures suivant leur administration, pour atteindre une efficacité de 99,6 % après huit heures. Le lotilaner peut donc être utilisé pour soulager rapidement l'irritation due à la présence d'infestations par les puces.
